# Vitiligo Masked by Steroid-Induced Rosacea-Like Dermatitis and Revealed by VISIA Ultraviolet Imaging: A Longitudinal Case Report

**DOI:** 10.7759/cureus.112942

**Published:** 2026-07-19

**Authors:** Yasir Radhi, Ali Almamoori

**Affiliations:** 1 Department of Dermatology, Al-Shaheed Al-Sadr General Hospital, Baghdad, IRQ; 2 Department of Dermatology, Baghdad Teaching Hospital, Baghdad, IRQ

**Keywords:** koebner’s phenomenon, steroid-induced rosacea-like dermatitis, topical corticosteroid misuse, visia imaging, vitiligo

## Abstract

Facial depigmentation may remain clinically unrecognized when obscured by persistent erythema associated with steroid-induced rosacea-like dermatitis (SIRD). We report a 42-year-old woman with Fitzpatrick skin phototype IV and a history of prolonged facial topical corticosteroid use who presented with persistent centrofacial erythema. Clinical examination was consistent with SIRD. Multimodal VISIA imaging revealed findings that were not appreciable under standard visible light: red-channel (RBX®) imaging demonstrated increased vascularity and telangiectasia, while ultraviolet imaging identified patchy depigmentation with a speckled pattern and perifollicular pigment preservation. Linear depigmentation corresponding to prior eyebrow tattooing suggested a Koebner phenomenon. Although advised to discontinue corticosteroids, the patient was lost to follow-up and returned three years later with persistent erythema and progression of depigmentation. Repeat ultraviolet imaging demonstrated an increased extent and partial coalescence of the depigmented areas. The longitudinal evolution, imaging features, perifollicular pigment preservation, and trauma-induced depigmentation were most consistent with evolving vitiligo coexisting with SIRD. Following corticosteroid withdrawal, topical tacrolimus 0.1% ointment was initiated. This case highlights how persistent facial erythema can mask progressive depigmentation and demonstrates the value of multimodal VISIA imaging, particularly red-channel and ultraviolet modalities, in detecting subclinical pigmentary changes, differentiating overlapping disease processes, and documenting disease progression.

## Introduction

Prolonged and inappropriate topical corticosteroid use on the face is a widely recognized cause of steroid-induced rosacea-like dermatitis (SIRD), a condition characterized by persistent centrofacial erythema, telangiectasia, papules, and a rebound phenomenon upon corticosteroid withdrawal [1,2]. The vascular and inflammatory changes of SIRD may substantially alter the clinical appearance of the face, potentially obscuring coexisting dermatoses and delaying their recognition.

Vitiligo is an acquired autoimmune depigmenting disorder resulting from the selective destruction of cutaneous melanocytes [3]. In individuals with darker skin phototypes, the contrast between affected and unaffected skin is typically more pronounced; however, when depigmentation is patchy or early and the background skin exhibits prominent erythema, the pigmentary changes may become visually indistinct under standard visible-light examination.

Noninvasive multimodal imaging platforms such as the VISIA Complexion Analysis System (Canfield Scientific, Parsippany, NJ, USA) offer complementary modes of skin assessment beyond conventional clinical examination. The system employs standardized white-light, cross-polarized, and ultraviolet imaging, with red-channel (RBX®) processing providing additional visualization of vascular features [4,5]. Specifically, ultraviolet imaging can enhance the visualization of variations in epidermal melanin distribution because of the absorption of ultraviolet radiation by melanin, thereby potentially facilitating the identification of areas of subclinical depigmentation [6].

We present a longitudinal case in which SIRD-related erythema clinically masked progressive facial vitiligo. Multimodal VISIA imaging, particularly the ultraviolet mode, was instrumental in detecting and documenting the evolution of subclinical vitiligo over a three-year interval, including evidence of the Koebner phenomenon at a prior tattoo site.

## Case presentation

A 42-year-old woman with Fitzpatrick skin phototype IV presented with a several-year history of persistent centrofacial erythema predominantly involving the cheeks and nose. She reported prolonged self-application of mometasone furoate 0.1% (1 mg/g), a medium-potency topical corticosteroid, to the face without a clear therapeutic indication. There was no reported personal or family history of autoimmune disease.

Clinical examination revealed diffuse centrofacial erythema with scattered inflammatory papules and telangiectasia, consistent with SIRD. No well-defined depigmented patches were appreciable under routine clinical lighting.

Multimodal facial imaging using the VISIA system was performed. Red-channel (RBX®) imaging demonstrated increased vascularity and prominent telangiectasia, particularly over the malar regions. In contrast, ultraviolet imaging revealed multiple patchy depigmented areas with a speckled appearance and relative perifollicular pigment preservation that were not clinically visible. Linear depigmented streaks corresponding to sites of previous eyebrow tattooing were also identified, suggesting a Koebner phenomenon induced by tattoo-related trauma.

The patient was counseled regarding SIRD, advised to discontinue topical corticosteroids, and informed that the imaging findings were suggestive of early vitiligo. However, she was subsequently lost to follow-up.

Three years later, she returned with worsening cosmetic concerns. Although centrofacial erythema remained the dominant clinical feature and depigmentation was still difficult to appreciate under visible light, repeat ultraviolet imaging demonstrated a marked increase in the extent of the depigmented areas, with partial coalescence of previously discrete foci, indicating disease progression.

**Figure 1:**
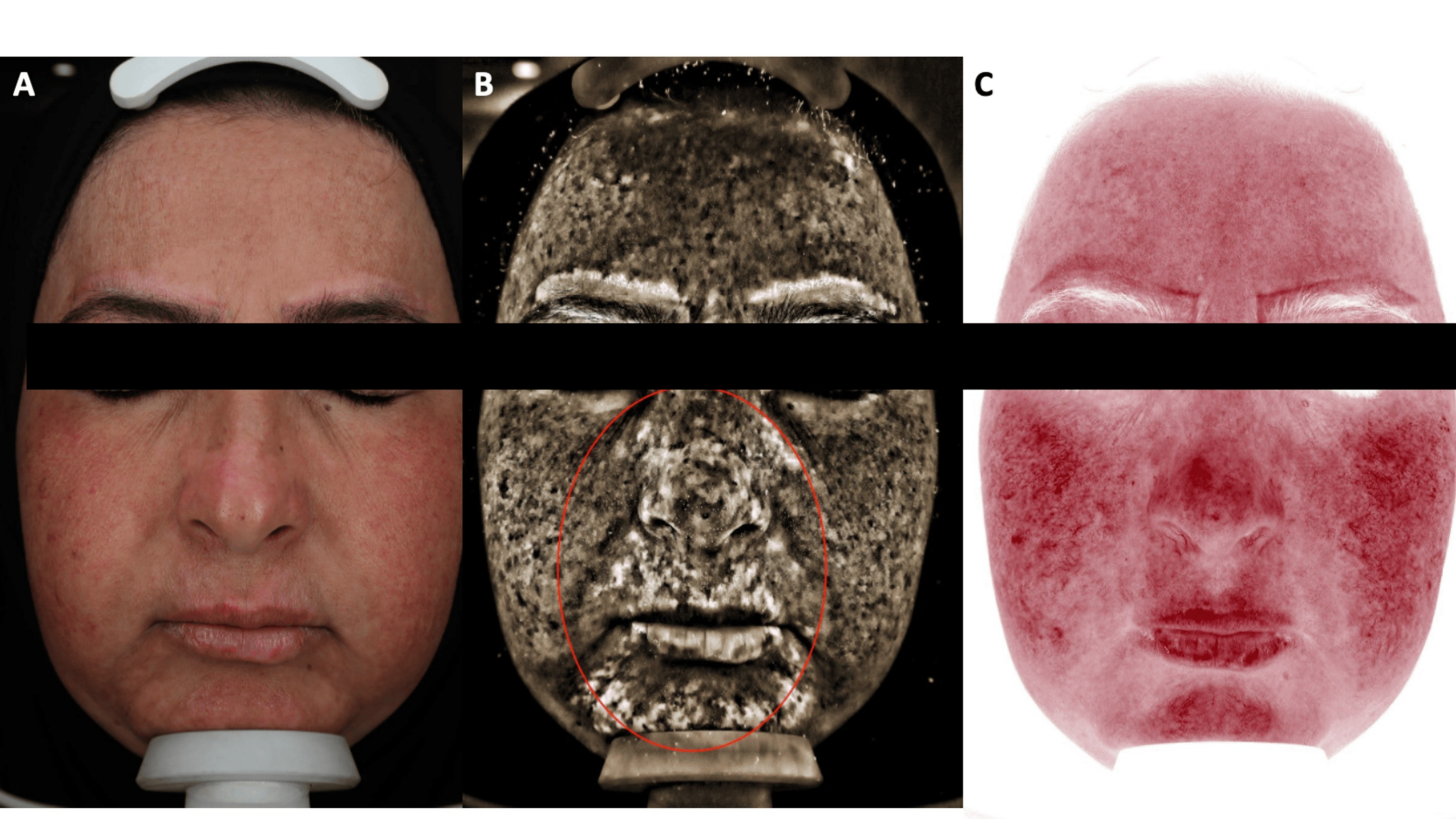
Multimodal VISIA imaging at initial presentation. (A) Standard clinical photograph demonstrating diffuse centrofacial erythema with scattered inflammatory papules and telangiectasia, consistent with steroid-induced rosacea-like dermatitis. No clinically apparent depigmented patches are visible under conventional lighting. (B) VISIA ultraviolet (UV) image revealing multiple patchy depigmented areas with a speckled appearance and relative perifollicular pigment preservation involving the central face (circled), findings that were not appreciable on routine clinical examination. Linear depigmented streaks corresponding to prior eyebrow tattoo sites are also evident, suggestive of a Koebner phenomenon. (C) VISIA red-channel (RBX®) image demonstrating increased vascularity and prominent telangiectasia, particularly over the malar regions and nose, highlighting the vascular component of steroid-induced rosacea-like dermatitis.

The progressive course over three years, perifollicular pigment preservation, koebnerization at tattoo sites, and the absence of features typical of steroid-induced hypopigmentation, which is generally reversible following corticosteroid discontinuation, supported a diagnosis of evolving non-segmental vitiligo coexisting with SIRD. Following confirmation of corticosteroid discontinuation, topical tacrolimus 0.1% ointment was initiated as a steroid-sparing treatment for facial involvement (Figures 1A-1C).

## Discussion

This case highlights a clinically important diagnostic challenge: persistent facial erythema secondary to topical corticosteroid misuse may effectively mask coexisting and progressive pigmentary pathology, particularly in patients with intermediate to darker skin phototypes. The combination of reduced epidermal melanin in depigmented areas and increased dermal vascularity in SIRD can diminish the visible contrast between affected and unaffected skin, rendering depigmentation invisible under standard clinical lighting.

The VISIA Complexion Analysis System addresses this diagnostic limitation by capturing distinct components of facial skin pathology through multiple illumination modes [4-5]. In this case, the red-channel (RBX®) images objectified the vascular component of SIRD, confirming telangiectasia and increased vascularity, while the ultraviolet mode revealed subclinical depigmentation that was entirely occult under visible light. This dissociation of vascular and pigmentary signals was the pivotal diagnostic insight afforded by multimodal imaging.

Ultraviolet imaging detects areas of reduced epidermal melanin by exploiting the preferential absorption of UV radiation by melanin; hypopigmented or depigmented areas therefore appear fluorescent or with heightened contrast on UV photography [6]. Prior studies using UV-mode dermoscopy have demonstrated that progressive (unstable) vitiligo characteristically shows perifollicular pigment preservation and poorly defined borders, while stable vitiligo exhibits sharply demarcated margins with perifollicular depigmentation [7-9]. The imaging findings in our patient, speckled hypopigmentation with preserved perifollicular pigment, were accordingly consistent with active, progressive vitiligo rather than a stable or resolving process.

The identification of linear depigmentation at the sites of prior eyebrow tattooing provides additional diagnostic support for vitiligo through the Koebner phenomenon. The Koebner phenomenon, defined as the development of disease-specific lesions at sites of cutaneous trauma in previously uninvolved skin, is a well-recognized feature of vitiligo, observed in approximately one-third of affected patients and considered indicative of disease activity. van Geel N et al. specifically identified tattoos among the provoking traumatic stimuli capable of inducing koebnerization in vitiligo [7]. The linear, site-specific morphology of the depigmented streaks at the tattoo sites in our patient is consistent with type 2B koebnerization (linear, artifactual lesions based on clinical examination) [8]. This finding is not a feature of steroid-induced hypopigmentation, which does not exhibit the isomorphic response, and therefore further differentiates the two conditions.

Steroid-induced hypopigmentation results from the suppressive effect of topical corticosteroids on melanocyte activity, producing reversible, non-progressive pigmentary changes that typically resolve upon cessation of corticosteroid use [1,2]. In contrast, the depigmented areas in our patient demonstrated progressive expansion and coalescence over three years, a course incompatible with simple steroid-induced suppression and consistent with the variable, often progressive natural history of vitiligo [3].

Topical tacrolimus 0.1%, a calcineurin inhibitor with immunomodulatory properties, was selected as first-line treatment following corticosteroid withdrawal. Its mechanism involves inhibition of T-cell activation and downstream pro-inflammatory cytokine transcription, thereby reducing the autoimmune-mediated melanocyte destruction central to vitiligo pathogenesis [10]. Topical calcineurin inhibitors are particularly suited to facial vitiligo, both because of the favorable response rates in this anatomical region and because they carry no risk of cutaneous atrophy, a critical consideration in a patient with pre-existing steroid-related skin changes [10,11]. A multicenter randomized controlled trial demonstrated that twice-daily tacrolimus 0.1% ointment produced superior repigmentation compared with vehicle in facial vitiligo over 24 weeks, and a systematic review and meta-analysis of 46 studies confirmed that face and neck lesions respond more favorably to topical calcineurin inhibitors than other body sites, with at least moderate repigmentation achieved in 57.5% of such lesions [11].

This case demonstrates that in complex facial dermatoses where multiple pathological processes coexist, clinical examination alone may be insufficient for complete characterization. The integration of multimodal imaging, and the VISIA ultraviolet mode in particular, into the clinical assessment workflow enables the detection of subclinical pigmentary changes, supports diagnostic differentiation, and provides objective documentation of disease progression over time.

This report describes a single case and is subject to the inherent limitations of case-report methodology. Histopathological confirmation of vitiligo was not obtained; the diagnosis rests on clinical and imaging criteria, including the characteristic pattern of perifollicular pigment preservation, longitudinal progression, and koebnerization. Laboratory evaluation for associated autoimmune conditions was not performed at either presentation. Additionally, loss to follow-up between visits precluded documentation of interim disease evolution and management. The absence of patch testing or provocation data limits the characterization of the relative contributions of corticosteroid misuse and evolving vitiligo to the overall clinical picture. The generalizability of the imaging findings is limited to settings with access to the VISIA platform or equivalent multimodal facial imaging systems.

## Conclusions

Vitiligo may remain clinically occult when obscured by steroid-induced erythema. In this longitudinal case, VISIA ultraviolet imaging was critical in identifying patchy facial depigmentation with perifollicular pigment preservation, detecting a Koebner phenomenon at a tattoo site, and documenting progressive disease expansion over three years, all features that were invisible under standard visible-light examination. Early and accurate recognition of coexisting pigmentary pathology in patients with SIRD-related erythema may prevent diagnostic delay and allow the timely initiation of appropriate therapy.
